# The Norwegian national project for ethics support in community health and care services

**DOI:** 10.1186/s12910-016-0158-5

**Published:** 2016-11-08

**Authors:** Morten Magelssen, Elisabeth Gjerberg, Reidar Pedersen, Reidun Førde, Lillian Lillemoen

**Affiliations:** Centre for Medical Ethics, Institute of Health and Society, University of Oslo, Pb. 1130 Blindern, N-0318 Oslo, Norway

**Keywords:** Clinical ethics, Clinical ethics support, Community care, Ethics reflection

## Abstract

**Background:**

Internationally, clinical ethics support has yet to be implemented systematically in community health and care services. A large-scale Norwegian project (2007–2015) attempted to increase ethical competence in community services through facilitating the implementation of ethics support activities in 241 Norwegian municipalities. The article describes the ethics project and the ethics activities that ensued.

**Methods:**

The article first gives an account of the Norwegian ethics project. Then the results of two online questionnaires are reported, characterizing the scope, activities and organization of the ethics activities in the Norwegian municipalities and the ethical topics addressed.

**Results:**

One hundred and thirty-seven municipal contact persons answered the first survey (55 % response rate), whereas 217 ethics facilitators from 48 municipalities responded to the second (33 % response rate). The Norwegian ethics project is vast in scope, yet has focused on some institutions and professions (e.g., nursing homes, home-based care; nurses, nurses’ aides, unskilled workers) whilst seldom reaching others (e.g., child and adolescent health care; physicians). Patients and next of kin were very seldom involved. Through the ethics project employees discussed many important ethical challenges, in particular related to patient autonomy, competence to consent, and cooperation with next of kin. The “ethics reflection group” was the most common venue for ethics deliberation.

**Conclusions:**

The Norwegian project is the first of its kind and scope, and other countries may learn from the Norwegian experiences. Professionals have discussed central ethical dilemmas, the handling of which arguably makes a difference for patients/users and service quality. The study indicates that large (national) scale implementation of CES structures for the municipal health and care services is complex, yet feasible.

**Electronic supplementary material:**

The online version of this article (doi:10.1186/s12910-016-0158-5) contains supplementary material, which is available to authorized users.

## Background

Over the last 20 years, clinical ethics support (CES) has become widespread in hospitals in Europe, including in Norway. Clinical ethics committees, ethics consultants and ethics rounds are typical CES structures. Such services have been shown to be helpful in the identification and handling of ethical dilemmas, and have received positive evaluations [1–8].

However, the development of ethics support in community health and care services (e.g. within nursing homes, home care services, local public health centres and among general practitioners) has been much sparser than within hospitals and other specialized medical care [9, 10]. This is surprising since there is little reason to believe that the need is any less in community services [9, 11–13]. Many of the users of community health services have multiple, long-lasting and severe diseases, as well as reduced decision-making capacity. Many nursing home patients, for example, suffer from dementia and various chronic diseases, and have complex and extensive health care needs. In long-term care settings, ethical challenges may be embedded in daily care. Thus, those who provide community health and care services face many and complex ethical issues [13, 14]. At the same time, the available resources, such as economic resources and professional training, are more limited. Many workers are unskilled and work is often performed alone.

### International research on ethics support in community healthcare

Healthcare professionals in community care experience many and important ethical dilemmas [9, 13, 15, 16]. Van der Dam et al. reviewed the literature on ethics support in institutional elderly care and identified 60 papers [10]. The earliest studies were from North America, due to the fact that ethics support was first developed there; several European countries are represented among the newer studies. The review identified four categories of ethics support mechanisms: institutional bodies, such as ethics committees; analytical tools to assist professionals; educational programs and moral case deliberation; and written documents and policies. The authors conclude that ethics support has gradually become ‘more outreaching and proactive, aiming to qualify professionals to integrate ethics in daily care processes [10].’ Furthermore, ‘[t]he approaches in clinical ethics support have become more diverse, more focused on everyday ethical issues and better adapted to the concrete learning style of the nursing staff’ [10].

The review does not detail the extent to which CES structures have been implemented; indeed, it is our impression that in most countries, CES implementation in community health and care services has typically not been comprehensive, but patchy and dependent on local initiatives. The scarcity of published reports on larger-scale implementations of CES in community care indicates that such implementations have hitherto been rare.

### Systematic ethics support in Norwegian municipalities

The aims of this article are, firstly, to present the Norwegian national project for CES in community services (present section); secondly, to characterize the ethics activities that the project gave rise to in the municipalities (the remainder of the article).

Norway has a population of 5.2 million. Norway’s 428 municipalities are responsible for primary health and care services for their populations (including elderly care, home-based care, sheltered housing/assisted living facilities, mental health and substance abuse care, school health services, maternal-child and adolescent health stations, primary care physicians). The average municipality has a population of 12,000. However, due to large areas being sparsely populated many municipalities are much smaller; 235 municipalities have fewer than 5,000 inhabitants. Health care in Norway is mainly public.

In Norway, a project to increase ethical competence in community health and care services through working systematically with ethics (henceforth “the ethics project”) ran from 2007 to 2015. The project was outlined in a 2006 Ministry of Health and Care Services report approved by Parliament, [17] and as part of an agreement between the government and the municipal sector on quality improvement in health and care services.

In these and related documents it was maintained that the level of competence within municipal health and care services was often low with many unskilled employees, that service recipients were often frail, and that there was a great need to improve competence in ethics among employees. Specifically, the government wanted the ethics project to”contribute to basic competence in professional ethics for employees in health and care services and develop models for embedding ethics work organisationally in the municipal context” [17]. Efforts to improve ethics competence were perceived as part of a strategy to improve service quality and professional competence in the care services overall.

The large-scale national ethics project was set up and headed by the Norwegian Association of Local and Regional Authorities (KS) [18]. As of 2013, KS provided 3.2 full-time positions in the project [19]. A steering group was set up with representatives from KS, the Ministry of Health and Care Services, labour and professional organizations, and the Centre for Medical Ethics (CME) at the University of Oslo.

All municipalities were invited to take part in the project. In order to be admitted to the project the municipality was required to have a project plan, a local project manager and the approval of municipal authorities. Municipalities were expected to report on their progress to the KS project management yearly.

**Fig. 1:**
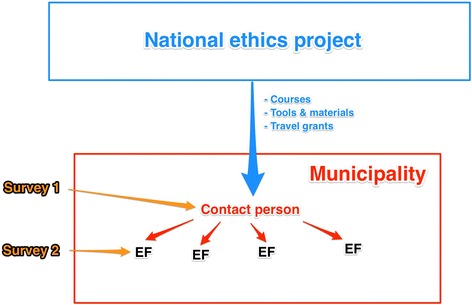
Surveys to stakeholders in the ethics project. For survey 1, the national project’s municipal contact persons were surveyed. In survey 2, respondents were ethics facilitators (EF) who were in charge of carrying out the actual ethics activities in their municipal health and care institutions

Municipal representatives were invited to a 2-day startup seminar where ethical reflection and the ethics project were introduced and practical exercises in ethics reflection performed. At later stages in a municipality’s project period representatives would be offered courses in facilitating ethics reflection, and regional and national conferences and courses were held. To some extent municipalities also received supervision and support from KS locally. The municipal stakeholders surveyed in the study are sketched in Fig. 1.

From the outset, participating municipalities were encouraged to develop local projects, selecting their own initiatives and developing their own implementation strategies [18]. From 2010 onwards, however, the project promoted ethics reflection groups (ERGs, to be described below) as a preferred ethics activity. The national ethics project contributed to the ethics activities in the municipalities mainly by offering national and regional courses in ethics reflection methods, by supplying travel grants, and by developing tools and material, such as books, pamphlets and ethics reflection cards (see Table 1). While KS was tasked with running the project itself and with the main responsibility for ethics training, the Centre for Medical Ethics contributed to training and carried out evaluation research.

**Table 1:** Some common methods for ethical reflection

CME (Centre for Medical Ethics) 6-step model: This is a sequential structure for ethical deliberation about actual clinical dilemmas in which the facts of the case, ethical problems, stakeholder interests, relevant ethical features and laws, and available courses of action are identified and discussed [27].
Other discourse ethics models: E.g., “yes” or “no” as responses to the ethical question are written on each flipchart/blackboard, as are stakeholders and their interests, values and norms. The reflection process involves an assessment of the advantages and disadvantages of the two sides.
Ethics cards: Cards presenting statements, questions, pictures, or short descriptions of situations that contain ethical challenges are taken as point of departure for group discussions.
“Fish bowl” method: A sequential conversation, where participants sit in a circle and respond in turn by referring to the previous statements: “When you say…, (looking at one participant), I think …, (turning to the next participant)”, handing the statement to the next person in the ring.
“Traffic light” method: Red, green and yellow “traffic lights” are markers to signal that you agree (green), disagree (red) or are uncertain (yellow) about the ethical question and the proposed solution.

Municipalities applied and were admitted step-wise throughout the project period. As of early 2015, 241 of Norway’s 428 municipalities had been included in the project and a great variety of measures had been instigated. In an international context, this project is, we believe, unique in terms of focus, size and time span.

### A national-scale evaluation study

Previous, smaller-scale studies of the Norwegian ethics project have indicated a large variety in organization, activities and methods of the ethics initiatives across municipalities [19–21]. In some municipalities, the ethics activities have ceased. In most municipalities, ethics activities consist of discussing actual dilemmas experienced in the professionals’ practice, [22] yet only a minority use structured methods for ethical deliberation [19]. The present study is the first to survey the Norwegian ethics project on a national scale, where we ask what the municipal ethics activities have consisted of, and what consequences they have had for the services and the professionals.

In the present paper we map out the scope and participants of the municipal projects, describe the ethical issues that have been discussed, and detail the types of ethics activities and methods employed. In a companion paper we assess the significance of the ethics project for the services, and explore facilitating and inhibiting factors [23].

As part of the project, a few municipalities have established clinical ethics committees; nine are presently operational. These committees were not assessed in the present study, but are being evaluated independently.

## Methods

Data were collected through two online surveys, the first to the person responsible for the ethics project in each municipality (henceforth the “municipal contact person”), the second to those leading the actual ethics activities in their institutions (henceforth the “ethics facilitators”). The questions were chosen based on our prior research on and impressions of the municipal ethics activities, especially from the municipalities’ own written reports. Both questionnaires underwent pilot testing. An English translation of the questionnaires is available as an Additional file 1.

The Norwegian ethics project is quite complex (see Fig. 1). In previous smaller-scale evaluations [19] we found that municipal contact persons, who one would think would have detailed knowledge of the ethics project in their municipality, in many cases had only superficial knowledge of the actual activities in the institutions. For this reason, we now wanted to survey ethics facilitators as well. Our intention was for the two surveys to different groups of project stakeholders to supplement one another, in order to provide a fuller picture of the ethics activities.

In the first survey (Jan.-Mar. 2015), the person who was listed as the contact person for the ethics project for each municipality was invited by email to answer an online questionnaire. The questionnaire asked for information about the municipality, the ethics activities and participants; the perceived usefulness of the project; and the number of ethics facilitators the municipality had appointed and educated, along with their email addresses.

Next, in the second survey (Apr.-Sep. 2015) each ethics facilitator whose email address had been provided through survey 1, was invited by email to answer a second online questionnaire. Here, respondents were asked to detail the ethics activities they had contributed to, the methods used, and their experiences with these. Respondents were also asked about promoters and inhibitors for ethics activities.

For both surveys, answers were typically sought in simple graded formats tailored to each question, such as rating occurences as “frequently”, “sometimes”, “seldom” or “never”. Non-responders were sent two emails as reminders. In order to assess characteristics of non-responding municipalities we contacted nine municipalities that had not responded to survey 1 for brief telephone interviews.

Simple descriptive statistical analyses were performed in IBM SPSS Statistics version 22.

## Results

### Survey 1: respondents and services involved

For survey 1, invitation emails were sent to 242 contact persons in 241 municipalities (in one large municipality the ethics project was divided in two, for municipal and private-run services respectively); 137 answers were received, yielding a 57 % response rate. Respondents detailed that a sum total of 2515 ethics facilitators had been trained/appointed in their municipalities; we received 662 functioning email addresses to ethics facilitators in 63 municipalities.

Ethics activities were most prevalent in nursing homes (78 %), home-based services (74 %) and sheltered housing (assisted living facilities; 63 %). In somewhat less than half of the municipalities, ethics activities also took place in mental health care (45 %) and substance use care (35 %). Ethics activities seldom reached health stations for child and adolescent health (16 %), primary care physician services (12 %) or school health services (11 %). Of note, many contact persons were unsure about the prevalence of ethics activities in various municipal services.

Municipal contact persons were also asked whether the municipality had arranged ethics seminars open to all employees in the health and care sectors. Nineteen municipalities (14 %) had done so often, 97 (71 %) sometimes, whereas 15 (11 %) had not (6 (4 %) did not know/unanswered).

### Survey 2: respondents

For survey 2, invitation emails were sent to the 662 ethics facilitators whose email addresses were received through the first survey; 217 answers from ethics facilitators in 48 municipalities were received, entailing a 33 % response rate. Of these respondents, 88 % were presently active as ethics facilitators, 61 % monthly or more often. Ethics facilitators were most commonly nurses (47 %), nurses’ aides (20 %), or social educators (14 %). Nineteen percent of the facilitators were department/ward leaders.

One hundred and fifty-two (70 %) had received training locally in the municipality, whereas 141 (65 %) had participated in regional/national courses (e.g., arranged by KS, CME or university colleges). Slightly less than half (47 %) had received (at least some) supervision in the role as ethics facilitator.

### Survey 2: participants, forms and methods of ethics activities

The professions most commonly involved in the ethics activities were nurses’ aides, nurses and unskilled workers (Table 2). Patients/users and next of kin were seldom involved, as were physicians. (Survey 1 provided some additional information about the physicians: when physicians had been involved it was either through participation in ethics reflection groups (7 of 137), through contributing to planning the municipal ethics project (3) or through involvement in teaching or seminars (3)).

**Table 2:** Whether and how often various professions/stakeholders had been involved in ethics activities. *N* = 217

Profession	Frequently	Sometimes
Nurses’ aides	152 (70 %)	28 (13 %)
Nurses	118 (54 %)	27 (12 %)
Unskilled workers	112 (52 %)	51 (24 %)
Dept./ward leaders	87 (40 %)	58 (27 %)
Social educators	76 (35 %)	31 (14 %)
Occupational therapists	23 (11 %)	18 (8 %)
Physiotherapists	10 (5 %)	18 (8 %)
Physicians	4 (2 %)	7 (3 %)
Next of kin	3 (1 %)	10 (5 %)
Patients/users	2 (1 %)	12 (6 %)

Ethics facilitators were asked about *which forms* the ethics activities had taken at their workplace and *which methods* were used for ethics reflection. Table 3 lists the most common forms of ethics activities. Ethics reflection groups (ERGs), wherein employees gather to (typically) discuss an actual case from their own department, were by far the form most commonly employed. Of the 217 respondents, 186 stated having had experience with ERGs; 128 (59 % of all) stated that ERGs were *currently* regularly performed at their workplace. In addition to the ethics activities listed in the questionnaire and in Table 3, some respondents also stated that employees would be convened for ad hoc ethical discussions when the need arose.

**Table 3:** Ethics activities attempted and currently in use

Ethics activity	Experience with the activity (% of all respondents, *N* = 217)	Activity *currently* performed at workplace (% of respondents with experience with the activity)
Ethics reflection groups	186 (86 %)	128 (69 %)
ER as part of personnel meetings	113 (52 %)	67 (59 %)
ER as part of report meetings	95 (44 %)	43 (45 %)
ER as part of theme day/seminar	71 (33 %)	22 (31 %)
Ethics café/lunch	43 (20 %)	24 (56 %)

*ER* ethics reflection, “*report meetings*” brief meetings in connection with shift changes

Ethics facilitators reported that ERG sessions typically lasted 30–90 min (median 64 mins; range 15–180 mins.), whereas ethics sessions as part of report meetings or personnel meetings were briefer (medians 18 mins and 35 mins respectively).

Ethics facilitators were also asked about their experiences with various *methods* or structures used for ethics discussions in the different ethics activities (Table 4). The most common methods presented in the training of facilitators are detailed in Table 1. Most had experiences with unstructured reflection, the CME (Centre of Medical Ethics) 6-step model or ethics cards. Of these, the CME model was perceived as well suited by the highest proportion (69 %), but other models also received positive or mixed evaluations. Thirty respondents (14 %) had experiences with other methods not listed in the questionnaire.

**Table 4:** Experiences with various methods for ethics discussions and their suitability

Methods	Experience with the method (% of all respondents, *N* = 217)	Method *currently* in use at workplace (% of respondents with experience with the activity)	Method thought to be well suited (% of respondents with experience with the activity)
Unstructured/free reflection	167 (77 %)	133 (80 %)	84 (50 %)
CME 6-step model	125 (58 %)	107 (86 %)	86 (69 %)
Ethics cards	107 (49 %)	62 (58 %)	63 (59 %)
Other discourse ethics model	51 (24 %)	26 (51 %)	24 (47 %)
”Traffic light” method	39 (18 %)	13 (33 %)	14 (36 %)
”Fish bowl” method	30 (14 %)	11 (37 %)	17 (57 %)^a^

^a^: Indicated as “not suited” by 11 respondents (37 % of respondents with experience with the method.)

### Survey 2: ethical dilemmas discussed

Ethics facilitators were asked about the ethical dilemmas discussed (Table 5). Of the ten topics specified in the questionnaire, eight were discussed “often” or “sometimes” according to a majority of respondents. In addition to the topics listed, 29 respondents stated other areas of concern; here, the topic mentioned most often was ethical issues about handling challenging patient/user behaviour.

**Table 5:** Frequency of discussion of ethical issues (survey 2)

Ethical challenge/topic	Often	Sometimes	Seldom/never	Do not know	N
Patient autonomy	112 (54 %)	79 (38 %)	10 (5 %)	5 (2 %)	206
Decision-making competence	71 (35 %)	90 (45 %)	35 (17 %)	5 (2 %)	201
Cooperation with next of kin	63 (31 %)	110 (54 %)	28 (14 %)	4 (2 %)	205
Quality and competence in the services	58 (28 %)	120 (59 %)	23 (11 %)	3 (1 %)	204
Confidentiality	56 (28 %)	105 (52 %)	36 (18 %)	5 (2 %)	202
Scarcity of resources/personnel^a^	56 (27 %)	92 (45 %)	50 (24 %)	7 (3 %)	205
Use of coercion^b^	54 (27 %)	84 (42 %)	54 (27 %)	7 (4 %)	199
Work environment/tolerance for criticism	48 (24 %)	102 (50 %)	52 (25 %)	2 (1 %)	204
End-of-life ethics^c^	27 (14 %)	62 (31 %)	97 (49 %)	12 (6 %)	198
Challenges with different cultures	20 (10 %)	62 (31 %)	105 (53 %)	11 (6 %)	198

^a^:More often discussed in nursing homes, least often in mental health care

^b^:More often discussed in nursing homes and sheltered housing, less often in other institutions

^c^:Primarily a topic in nursing homes (82 % often or sometimes), seldom in other institutions

### Ethics activities in non-responding municipalities

In order to assess non-response bias, we contacted nine randomly selected municipalities who had participated in the national ethics project but whose contact person did not answer survey 1. Of these, four had experienced a stable or increased level of activity. Regular ethics activities were still conducted in five of the nine municipalities.

## Discussion

### Strengths and limitations

The endeavour of evaluating this large-scale national project is difficult, as the local organization of the ethics activities and the municipalities themselves are quite heterogeneous. Surveys addressing two different levels of the municipal organization were employed to achieve a comprehensive overview. Still, neither the employees who have participated in the ethics activities nor the patients/users have been reached by the surveys; their experiences should also be studied.

The two surveys both have a large number of responders with a nationwide distribution and with representation from small, medium and large, urban and rural municipalities. However, in survey 2 only 48 of the 241 participating municipalities are represented. The low response rate of survey 2 is, we believe, partly explained by the fact that many professionals work in a patient- and user-centred setting in which computers and work emails are used only sporadically; some ethics facilitators may not have registered the invitation email. Still, the low response rate means that a possible non-response bias, in which facilitators with more positive experiences were more likely to participate in survey 2, cannot be excluded. The non-responder survey indicates that responses to survey 1 are representative of the municipalities participating in the ethics project as a whole.

### Distribution of ethics support

The study indicates the complexity and the large scope of the Norwegian ethics project. Health and care institutions in about half of all Norwegian municipalities have commenced CES activities, a large number of local ethics facilitators have been trained, and professionals have participated in diverse ethics activities. In a project implementation perspective this is an impressive uptake.

However, the surveys show that the initiatives have not reached the entirety of the health and care sectors. Ethics activities are most prevalent in nursing homes and home-based services, to a lesser extent in mental health and substance use care, whereas it has reached child and adolescent healthcare and general practitioners only in exceptional cases.

The project has involved physicians only sporadically. We perceive this as an important shortcoming, for several reasons: First, physicians in the municipal health services (e.g., general practitioners, nursing home physicians) experience difficult, yet significant, ethical challenges with which CES could be helpful. Second, clinical ethical dilemmas in community care are typically complex and multidisciplinary in nature. All employees involved should contribute their professional insights and perspectives. Deliberations and the invention of appropriate solutions will be hampered when key stakeholders such as the physicians do not participate. Physician organizations such as The Norwegian Medical Association were not involved in the planning of the national ethics project, and this we perceive as unfortunate.

Similarly, the study shows that there is much room for improvement in including other key stakeholders, namely, patients and relatives, in the ethics activities. A key aim of the ethics project was to promote user participation. It is therefore somewhat paradoxical that they have so seldom been involved in the ethics activities. Studies have shown that it can be challenging, yet still feasible and beneficial, to include patients and next of kin in ethics deliberations when appropriate [7, 24].

### Organization of activities

The ERG was the most commonly used forum for ethics discussions. This was also the form attempted that most institutions continued to use. Significantly more time is set aside for the ERG than for other common ethics forums, such as ethics reflection as part of personnel or report meetings. Although professionals in the Norwegian health and care services are often pressed for time, time may be a prerequisite to allow for sufficiently broad and deep discussions of ethical dilemmas experienced by the professionals themselves in their interaction with patients/users. This would be difficult to achieve in the briefer ethics forms.

A large majority of municipalities had also arranged ethics seminars, a low-threshold way of bringing ethical competence, perspectives and awareness to the professionals.

Many municipal contact persons were unsure whether ethics activities were presently carried out in segments of the municipal services, and many did not provide us with email addresses for the municipality’s ethics facilitators. This may indicate a lack of sufficient overview of municipal ethics activities that could hamper important communication, supervision and network formation needed to maintain and develop the ethics initiatives.

### Ethical challenges in community health and care

The ethical issues named in the questionnaire (Table 5) were chosen based on previous research findings about common challenges in Norwegian community care [13, 14]. The present findings indicate that ethical dilemmas in these areas are common. Professionals appear to consider these topics as significant, in that the topics are often brought up for discussion in the ethics activities. Arguably, all the common ethical issues are closely connected to the quality of the services. This points to a genuine need for CES activities in this area of health and care services: the ethics activities deal with complex and central matters, the handling of which most likely makes a difference for patients/users and next of kin. Apparently, the most common ethical challenges are also encountered in similar services in other countries, as other research indicates [9, 25, 26].

There were differences between institution types as to which topics were discussed. Scarcity of personnel and resources was a more common topic in nursing homes than in other institutions, perhaps indicating that nursing home employees are more pressed for time. Coercion was primarily discussed in nursing homes and sheltered housing, apparently less often being morally problematic in home-based services.

### Suitability of ethics deliberation methods

The CME 6-step model and free reflection were the two most prevalent methods for structuring the ethical deliberations, and are more often continued than the other methods tried out in the activities. Free reflection may appear attractive because it does not require proficiency in structured models and does not constrain discussion. The CME 6-step model may be well suited because it provides a relatively simple structure which fosters the articulation of diverse morally relevant considerations, while being geared towards inventing constructive solutions [27].

One interpretation of why the CME method and free reflection are more prevalent is the following: Whereas the simpler methods such as ethics cards, “fish bowl” and “traffic light” models are perceived as accessible and undemanding introductions to ethical discussions, concepts and dilemmas, these methods are not designed to discuss the actual, concrete and complex challenges experienced by the professionals in their daily practice. These methods may also be perceived as constraining the ethical discussion; [20] the “traffic light” model, for instance, encourages participants to take a stand but does not promote ethical reflection. Therefore, if the ethics activities are to be sustained over time, they will sooner or later have to shift their focus onto actual challenges – which may turn out to constitute a never-ending reservoir of cases for discussion. For this, a structured reflection method may be helpful [28]. It is also likely that ethics reflection has a greater impact on practice if the topics for discussion are actual dilemmas rather than general issues, values and moral principles.

## Conclusion

We have given an account of the Norwegian national project for community health and care services and reported the results of two surveys that describe the ethics activities that have been performed in the municipalities. The surveys show that ethics activities have dealt with central ethical dilemmas that are arguably important for sevice quality. The study indicates that large-scale/national implementation of CES structures for the municipal health and care services is complex, yet feasible.

## Additional file

Additional file 1: English translation of the two questionnaires used in the study. (DOCX 112 kb)
