# Supplementary material for: The Norwegian national project for ethics support in community health and care services
Source: BMC Med Ethics. 2016 Nov 8;17:70. doi: 10.1186/s12910-016-0158-5 (PMC5101716; doi:10.1186/s12910-016-0158-5)
Supplement: Additional file 1: — English translation of the two questionnaires used in the study. (DOCX 112 kb) [file 12910_2016_158_MOESM1_ESM.docx]

Magelssen et al.: **The Norwegian national project for ethics support in community health and care services**

**English translations of the two questionnaires**. Answer formats in parentheses

**Questionnaire 1**

(To contact persons in participant municipalities)

For which municipality do you answer the survey?

How many inhabitants does the municipality have?

*(under 5000; between 5000 and 20000; over 20000)*

Which part of the municipality’s ethics work do you oversee?

Consider the proposition: ”I have a good overview of the ethics work in the municipality’s health and social sector”

*(strongly agree; somewhat agree; somewhat disagree; strongly disagree; do not know)*

Which description fits best the development over time for the ethics project in your municipality?

*(the ethics program never started; the ethics project started, but has ended; the ethics project ended and had to be restarted; the ethics project gradually diminished since its start; the ethics project gradually increased since its start; the ethics project has remained the same size since its start; we have worked with ethics independently of KS’s project; do not know; none of the above)*

Has the municipality succeeded in making the ethics project last?

*(Yes, to a large degree; yes, to some degree; to a small degree/no; too early to tell; do not know)*

Have the employees in the following services got an offer of ethics reflection today, and to which extent?

*(Question matrix – vertical categories: nursing homes, home-based services, mental health care, substance abuse care, sheltered housing, school health services, health stations, physician services. Horizontal answer categories: have an offer to a large extent; have an offer to some extent; have no offer; do not know)*

Have the municipality’s physicians been involved in the ethics work?

*(To a large degree; to some degree; no/only sporadically; do not know)*

Can you describe the way in which physicians have been involved?

Have unskilled workers been involved in the ethics work?

*(To a large degree; to some degree; no/only sporadically; do not know)*

Has the municipality taught/held seminars in ethics for employees in the health and care sector as part of the ethics project?

*(Yes, often; yes, sometimes; no; do not know)*

Is your impression that the ethics project has received support from the following levels in the municipality? What significance has this support had?

*(Question matrix – vertical categories: support on political level; support on municipal leadership level; support on institution leader level; support on department leader level; support from employee representatives; support from employees. Horizontal answer categories: no support; the support has had great significance; the support has had some significance; the support has had little/no significance; do not know)*

In your opinion, what is the significance of the municipality’s ethics work for practice?

*(Very large significance; large significance; some significance; little significance; no significance; negative significance; do not know)*

In your view, has the municipality’s ethics work had sufficient:

*(Question matrix – vertical categories: economic resources; time; support; number of ethics facilitators; knowledge of ethics. Horizontal answer categories: to a large degree; to some degree; to a little degree/no; do not know)*

What kind of follow-up has the municipality’s ethics work received from KS?

*(Question matrix – vertical categories: ethics; practical execution of ethics reflection; help in receiving support on institution or department level; help in receiving support on municipality official level; organization/cooperation with development centres; organization/cooperation with college/university)*

Was the follow-up from the project leadership at KS sufficient?

*(yes; no)*

What kind of follow-up would you have wanted?

*(ethics knowledge; practical execution of ethics reflection; help in receiving support on institution or department level; help in receiving support on municipality official level; organization/cooperation with development centres; organization/cooperation with college/university; other – please explain)*

Have you cooperated with other municipalities through the ethics project?

*(yes; no)*

How important has this cooperation been for the results of the ethics project?

*(very important; important; not important; do not know)*

Do you want to cooperate with other municipalities about ethics reflection?

*(yes; no; do not know)*

Does the municipality have an overview of ethics facilitators, with the names and email addresses of these?

*(yes; partly; no; do not know)*

How many ethics facilitators have been trained in the municipality as part of the ethics project?

In which year did the municipality commence its ethics work?

*

**Questionnaire 2**

(To ethics facilitators in participant municipalities)

In which municipality do you work?

Are you active as ethics facilitator today?

*(Yes, several times a month; yes, monthly; yes, sporadically; no)*

Training as ethics facilitator in the ethics project. Please state which kinds of training you have received, and the degree to which this has been useful.

*(Question matrix – vertical categories: KS’s starting conference; ”Nordpå” course; KS’s basic course; Centre for medical ethics introductory seminar; ethics course at college/university; internal municipal teaching; other. Horizontal response categories: very useful; useful; of little use; have not received)*

Have you had running supervision in the role as ethics facilitator?

*(To a large degree; to some degree; to a little degree/no. If yes, from what kind of person (e.g., university teacher, nurse specialist) have you received supervision?)*

Which ethics activities have been attempted in your department/your departments? And to what degree have these been significant for practice?

*(Question matrix – vertical categories: ethics reflection groups; ethics reflection as planned part of report meetings; ethics reflection as planned part of personnel meetings; ethics reflection as planned part of staff seminar days; ethics cafe/ethics lunch; ethics cards; others. Horizontal response categories: great significance; some significance; little/no significance; uncertain significance; have not attempted)*

Which models for ethics deliberation have been attempted in your department(s), and to what degree do you consider the models as suitable?

*(Question matrix – vertical categories: ”Fish bowl” model; traffic light model; Centre of medical ethics model/6-step model; other discourse ethics model; ethics cards; free reflection/deliberation without any model; other. Horizontal response categories: well suited; somewhat suited; not suited; have not attempted)*

Which ethics activities are presently being conducted in your department?

*(ethics reflection groups; ethics reflection as planned part of report meetings; ethics reflection as planned part of personnel meetings; ethics reflection as planned part of staff seminar days; ethics cafe/ethics lunch; ethics cards; others (please specify))*

Which models for ethics deliberation are presently being used?

*(”Fish bowl” model; traffic light model; Centre of medical ethics model/6-step model; other discourse ethics model; ethics cards; free reflection/deliberation without any model; other (please specify))*

How often are ethics activities conducted in your department (or the department in which you are most often involved)?

*(Multiple times weekly; weekly; every other week; monthly; more seldom; at present no regular schedule)*

Is a written report produced from ethics reflection group meetings?

*(usually; sometimes; no)*

How long does a typical meeting of the ethics reflection group last?

When you have ethics reflection as part of report meetings, how much time is typically spent on this?

When you have ethics reflection as part of personnel meetings, how much time is typically spent on this?

Have the following groups been participants of ethics reflection:

*(Question matrix – vertical categories: physiotherapists; occupational therapists; nurses; social educators; nurse’s aides; unskilled workers; physicians; department managers; patients/users; next of kin; others. Horizontal response categories: not employed by us/not relevant; to a large degree; to some degree; only exceptionally; no; do not know)*

In your view, how significant has the ethics project been for the department’s practice (services)?

*(Very large significance; large significance; some significance; little significance; no significance; do not know)*

How significant has the ethics project been for your own practice as health professional?

*(Very large significance; large significance; some significance; little significance; no significance; do not know)*

To what degree do you think that the ethics project has resulted in the following consequences for your workplace?

*(Question matrix – vertical categories: better handling of ethical challenges; better service quality; better relations to patients/users and next of kin; less use of coercion; better employee cooperation; better work environment. Horizontal response categories: to a large degree; to some degree; to a little degree/no; do not know/not applicable)*

Has the ethics work led to other consequences that you want to mention?

Specify how often the following issues have been raised in ethics discussions you have been involved in

*(Question matrix – vertical categories: patient autonomy; decision making competence; use of coercion; cooperation with next of kin; work environment and disagreement/tolerance for criticism; end-of-life ethics; scarcity of resources or personnel; quality and competence in the services; challenges with different cultures; confidentiality. Horizontal response categories: often; sometimes; seldom/never; do not know)*

Any other topics that have occurred often?

Has the department succeeded in making the ethics work last?

*(yes, to a large degree; yes, to some degree; to a small degree/no; too early to tell; do not know)*

What do you think are the most important reasons for the ethics work not having lasted?

Has the ethics work in your opinion had sufficient:

*(Question matrix – vertical categories: time; number of ethics facilitators; ethics competence. Horizontal response categories: to a large degree; to some degree; to a small degree/no; do not know)*

In your opinion, has the department’s ethics work received support or resistance from:

*(Question matrix – vertical categories: the employees?; department manager?; institution’s leader?; municipal leadership?. Horizontal response categories: large degree of support; some support; little/no support; some resistance; large degree of resistance; do not know)*

Have you had contact and cooperation with other ethics facilitators in the municipality?

*(to a large degree; to some degree; no)*

Are the other ethics facilitators in your municipality currently active as ethics facilitators?

*(to a large degree; to some degree; to a little degree/no; do not know)*

Do you need the following in the work as ethics facilitator going forward?

*(Question matrix – vertical categories: ethics teaching; training in practical execution of ethics reflection; supervision; meeting points with other ethics facilitators (network). Horizontal response categories: to a large degree; to some degree; to a little degree/no; do not know)*

Your primary place of service is:

*(nursing home; home-based services; mental health care; substance abuse care; sheltered housing; other (please specify))*

What is your professional background?

*(nurse; nurse’s aide; occupational therapist; physical therapist; social educator; physician; other (please specify))*
